# The Growth and Scope of Voluntary Hospitals in London

**Published:** 1897-09-18

**Authors:** 


### The Institutional Workshop.

### THE GROWTH AND SCOPE OF VOLUNTARY HOSPITALS IN LONDON.

From a Correspondent.

### IX.—EARLY HOSPITAL TREATMENT.

An anonymous writer in 1708 gives a vivid, it may be feared too faithful, picture of the patient's lot before the establishment of the voluntary hospitals had set emulation to work to alleviate their sufferings. Under the title, "The Dispensarians are the Patriots of Great Britain," this writer urges the spread of home relief to the sick poor, and does not mince matters in depicting the drawbacks to hospital treatment. It will be noticed that he speaks of a hospital as a modern writer might now do of the casual ward—as the last and lowest resort. "The modern British neglect of, and cruelty to, the poor buries their own life under their ruins. The Poor, who stood in the front and broke the force of diseases invading you, are rudely destroyed by Apothecaries and the other empirics. Till the College Dispensary published their immense charity, they were inhumanly thrown into Hospitals. But your charitable and wise ancestors commanded the Hospitals to receive only the wounded and maimed from the Fleets and Armies, or the Casualties of the City." (It is not clear from whence he derived this notion.) "Many now perish in the rude and hazardous conveyance to, or depart soon after their arrival in, the hospitals. The noisome stench of the rooms from the excessive numbers (though most carefully cleansed) corrupts the air and poisons their blood, with the offensive dressings or burnings of the maimed and wounded. Their sleep is prevented by the inquietude and pains of the various diseases, the groans of the departed. The gross diet, not differing from that of the house of labour and correction, the common shop medicines only, and the rare attendance of the physicians and surgeons, will oblige the magistrates to restore them to the design of the institution. . . . The managers have had no regard to the remonstrance of the physicians; they conceive the great judgment of the apothecaries is infallible, and may pass in any hospital as in all the private families. They are affected with the limbs taken off, the cutting out of the stone, the burnings and trepannings, and the other manual operations, and are diverted from reflecting the latent causes of diseases, &c., may be preferred before the works of the hand."

Two serious indictments are here brought against the ordinary treatment of disease in the hospitals. First, that the attendance of the physicians and surgeons in charge was often perfunctory and often neglected; secondly, that surgical treatment attained undue prominence, and that the patients were, in fact, regarded as subjects for practice of a peculiarly painful description.

In the course of the eighteenth century, and due undoubtedly to the emulation provoked by the new voluntary hospitals, the first abuse faded completely into oblivion. Medical men contended eagerly for posts at these institutions, even when they carried no salary, and all the rising talent, all the growing interest in medical research and surgical skill was born and nurtured in these centres. Nominally, medical schools had long been attached to the endowed hospitals. It was not, however, until the end of the eighteenth century that a regular and complete course of study was afforded to the student. Dr. Herbenden, writing 1794, mentions as a novelty: "There has lately been established in several of the London hospitals a plan of courses of lectures in all branches of knowledge useful to the student in physic. This must make London a school of physic superior to any in Europe."

All this increased activity meant, of course, increased interest in the patients, vigilant study of cases presenting unusual features, and diligent attention to cures. The patients benefited all along the line, not only by the new interest awakened but by the variety consequent on the founding of institutions untrammelled by past traditions. Unfortunately, the zeal shown by the medical practitioners was spasmodic, and fluctuated according to the character of those immediately in charge. There was no organised supervision, and "hospital scandals" failed to form a topic in the eighteenth century solely for lack of a medium for proclaiming them to the public.

The second abuse exposed by the anonymous writer we have quoted continued almost unchecked throughout the century. The tendency to regard the hospital wards as a field for acquiring manual skill in operations was so strongly marked a feature in the hospital surgeon for many generations that it has hardly yet been dissociated from him in the popular imagination. Howard was deeply impressed by the misery inflicted by apparently needless operations performed on the single judgment of the operating surgeon. He strongly urged on all hospital authorities the enforcement of a standing order that no "amputation should ever take place till after a consultation of three medical gentlemen who shall be of unanimous opinion that it is absolutely necessary, and that there is no probability of effecting a cure without the use of knife and saw." Hospital surgeons seem to have comprised among their number some of the best and some of the worst specimens of humanity, according as the spectacle of continual suffering served to deepen or brutalise their nature. Few, perhaps, equalled the brutality of the celebrated surgeon Akenside, who "would order some of the patients on his visiting days to precede him with brooms to clear the way and prevent the patients from too nearly approaching him." This was in 1765. But few, again, reached the humane disposition of the gentle Percival, of Manchester, whose "Medical Ethics," written about the same time, represent the high water-mark of surgical skill and consideration for the patient. Truth to tell, surgery in the days before chloroform was a terrible profession, and one does not willingly linger over the pictures of the operating-room which have come to us from that period. Yet when all has been said that can be said of the horrors of anticipation, the callousness of bystanders and surgeon, the tortures of the patient, and the causes retarding recovery, it is certain that many lives were saved by these surgeons of old days, and that very many of them possessed a degree of dexterity in the exercise of their craft and were graced with a measure of success little short of marvellous when we remember the impediments under which they were compelled to labour. So much, at any rate, seems to be clear, that there was no reluctance on the part of patients, by the latter part, at any rate, of the eighteenth century, to enter the hospitals. Everywhere is the same cry; numbers turned away from the doors, every bed full, emergency beds only kept free with the greatest difficulty, and in every hospital, owing to the continual pressure, efforts in progress for providing further accommodation. The story of every voluntary hospital in London has been a record of continuous expansion.

Now, it is quite certain that unless a reasonable prospect of recovery were offered to the patients they would not have presented themselves as in-patients, bound, it must be remembered, to undergo at the will of the surgeon any operation he might believe to be necessary. The poor are very quick indeed to take fright, and they had ample opportunities, more than any others, for judging of the kind of treatment to which they would be exposed. Every former patient would be eager to relate all that he saw and heard while he was an inmate of the hospital, and, under the defective state of discipline which we have noted, nothing could take place that was not immediately known to all. And visitors were freely (it would seem too freely) admitted who could judge for themselves. It may be urged that the poor were driven to the hospitals from sheer destitution, but it has been shown that the actually destitute were in fact excluded from many of them by the fees on admittance and the security for burial exacted from all. There can be no reasonable doubt that the poor were attracted to the hospitals from the hope of relief, founded on the experience of friends who had been kindly treated, and of many undoubted cures. The defects, which are painfully apparent studied in the light of modern knowledge, were absolutely inoffensive to the majority, who found a standard of decency, comfort, and diet in the hospitals far superior to anything which their own homes could offer. There are absolutely no data for determining the quality of the nursing at this period. Nearly all the nurse's art to-day consists in the practice of such cleanliness as would then have been considered highly deleterious to the patient, and in the manipulation of appliances not invented till nearly a century later. The nurses were drawn from the same class as the patients, and as common sense, kindliness, and sense of duty are the monopolies of no age or class, it is perhaps not altogether just to attribute to all the sick attendants of the past the vices which were no doubt exhibited by a few among the number.

